# Gynecological Cancer Oncobiome Systematic Review

**DOI:** 10.3390/cancers17193227

**Published:** 2025-10-03

**Authors:** Tomasz Łatkiewicz, Karolina Rasoul-Pelińska, Krzysztof Kułak, Rafał Tarkowski, Anna Kułak, Iwona Puzio

**Affiliations:** 1 I Chair and Department of Oncological Gynecology and Gynaecology, Medical University of Lublin, 20-059 Lublin, Poland; karolina.rasoul-pelinska@umlub.edu.pl (K.R.-P.); krzysztof.kulak@umlub.pl (K.K.); rafal.tarkowski@umlub.pl (R.T.); 2 Department of Diagnostic and Microsurgery of Glaucoma, Medical University of Lublin, 20-059 Lublin, Poland; puzioan@gmail.com; 3 Department of Animal Physiology, Faculty of Veterinary Medicine, University of Life Sciences in Lublin, 20-950 Lublin, Poland

**Keywords:** endometrial cancer oncobiome, ovarian cancer oncobiome, cervical cancer oncobiome

## Abstract

**Simple Summary:**

This systematic review is about the oncobiome of three most common gynecological cancers. Modern treatment of cancer is based on immunology, specific genes or receptors. For example, type of treatment or survival in endometrial cancer is based on molecular classification. The next chapter of treatments will be based on the microenvironment of tumors. In the future, oncologists will probably be able to choose which element of the oncobiome can be used as a treatment. At the moment, we know that the environment of cancers is not sterile. In our systematic review we described the specific microenvironment of three most common gynecological cancer: ovarian cancer, cervical cancer and uterine corpus cancer.

**Abstract:**

**Objective**: The primary objective of this systematic review is to present current knowledge about the oncobiome of gynecological cancers. **Methods**: Our systematic review contains data about the oncobiome of uterine corpus cancer, ovarian cancer and cervical cancer. Articles about other gynecological cancers were excluded. **Results**: A total of 72 articles were included in our systematic review. In uterine corpus cancer, cervical cancer and ovarian cancer, representatives of bacteria, fungi, viruses and parasites can be found. The oncobiome of ovarian cancer is connected with the oncobiome of head and neck cancers. Our systematic review proved that the human papilloma virus is connected with ovarian and cervical cancer. Gut dysbiosis can be used as a marker of ovarian cancer. In cervical cancer, we found the difference between the microbiota of healthy patients and patients with cervical cancer. *Methylobacter*, *Robignitomaculum*, *Klebsiella*, *Micromonospora* and *Microbispora* have an impact on overall survival. The microbiome of uterine corpus cancer is more differentiated than in cancer-free samples. Chronic endometrial inflammation has an impact on endometrial microbiome. **Discussion**: Treatment of gynecological cancers is changing permanently. Chemotherapy, as a systematic treatment, is being left in the past. Modern methods of therapy are addressed to specific genes. In the past, researchers claimed that tumors are sterile. However, the newest research indicates that malignancies were found to have genetic fragments of pathogens, which can be used as vectors for medications or as markers for the detection of a specific malignancy. Three most common gynecological cancers are as follows: endometrial cancer, ovarian cancer and cervical cancer. Each of these has their specific microbiome, which can be used for oncological treatment. These discoveries create possibilities for new, efficient methods of treatment. This systematic review analyzes publications about the composition of the gynecological tumor microenvironment, correlation between microbiomes of different organs, the female reproductive tract and the microbiome of the female reproductive tract during malignancy. Moreover, we provide information on the influence of some pathogens on the treatment.

## 1. Introduction

Will further research into the cancer microbiome be decisive? At the moment, new studies are focusing on specific genes. Gynecological malignancies such as uterine corpus cancer, uterine cervix cancer or ovarian cancer have a characteristic microbiome. The tumor microenvironment plays an important role during carcinogenesis. Next generations of chemotherapy or immunotherapy will focus on elements of microbiome to treat patients. The efficiency of treatments depends on the composition of the gut microbiome, female reproductive tract flora or urinary tract microbiota. The vaginal microbiome plays an important role in cervical cancer because its dysbiosis initiates the process of dysplasia. Special pathogens can be used in the early detection of cancer. Immunotherapy, for example, dostarlimab in uterine corpus cancer, is an important method, because selected pathogens could induce immunoresistance; therefore, it is crucial to find out how to turn that effect around. In ovarian cancer it is significant to detect the disease in the early stage. Pathogens, which are an element of the tumor microenvironment, can be used for early diagnosis. More scientific studies are required, especially in uterine corpus cancer and cervical cancer to explore elements of the microbiome and tumor microenvironment. Authors of this review are considering that the oncobiome could be used both for diagnosis and treatment.

## 2. Materials and Methods

This review was conducted and is reported in accordance with the Preferred Reporting Items for Systematic Reviews and Meta-Analysis (PRISMA) guidelines. This systematic review includes studies and research, which provide valuable evidence and data. For the selection of articles, our team decided to include only original articles that focused on the study of uterine corpus cancer, ovarian cancer and cervical cancer. We checked how many patients took part in the study and how many times the article was cited. We decided to exclude articles that did not describe the oncobiome of uterine corpus cancer, ovarian and cervical cancer from the review. Articles which were not applicable to the main subject of the manuscript were excluded. We conducted a comprehensive literature search and critically evaluated the collected studies. We used the PubMed and Google Scholar engine for searching useful studies. We have chosen articles published between 1 January 2007 and 1 February 2023, and after review we decided to add 3 manuscripts: from 1999, 2024, and 2025. We used the search terms “ovarian cancer oncobiome”, “gynecological cancer oncobiome”, “cervical cancer oncobiome”, “cervical cancer tumor microenvironment”, “ovarian cancer tumor microenvironment”, “gynecological cancer bacterium”, “endometrial cancer microenvironment”, “cervical cancer oncobiome”, “endometrial cancer oncobiome” in the abstract and title. Additionally, cited articles in the main studies were used. This review contains information only from English-language articles. Studies were used only when describing data about the oncobiome of three most common gynecological cancers. Only data about three main gynecological cancers were extracted: ovarian cancer, cervical cancer and corpus uterine cancer. For the eligibility process, three authors (T.Ł., K.R.-P. and K.K.) alone screened the title and abstracts of all non-duplicated papers and did not include articles which did not match the set standards topic. The same three authors reviewed for a second time articles which were chosen after the first screening and decided which were perfect for our article. Discrepancies were discussed between the authors.

## 3. Results

**Figure 1:**
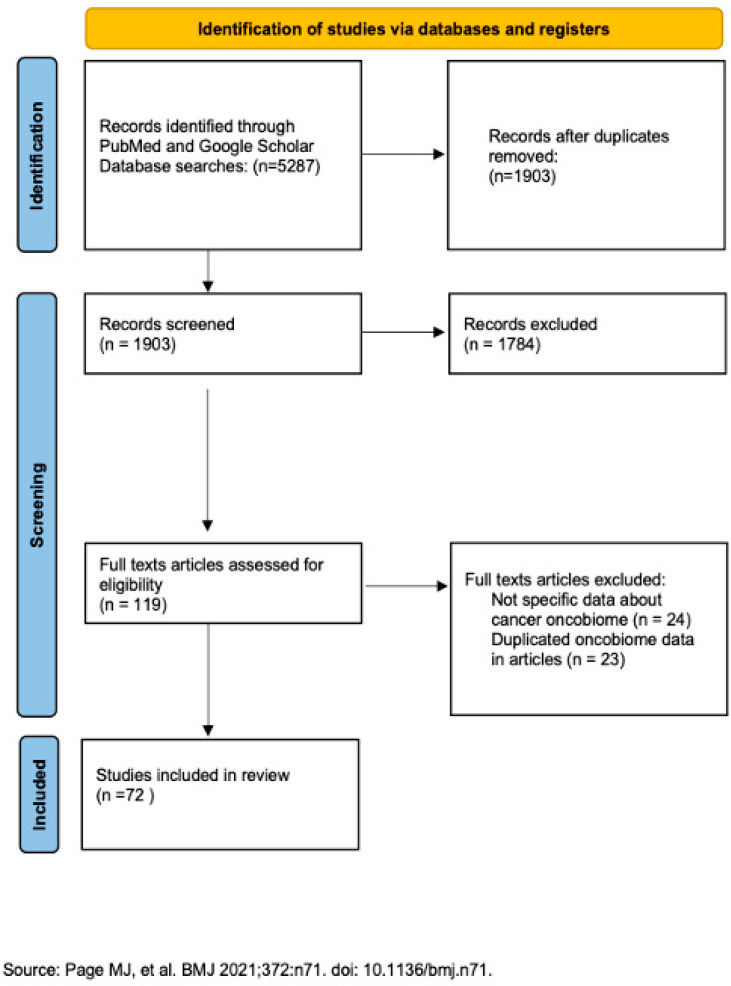
Preferred reporting items for Systematic Reviews and Meta-Analysis (PRISMA) flow diagram illustrating the study selection process [1].

**Figure 2:**
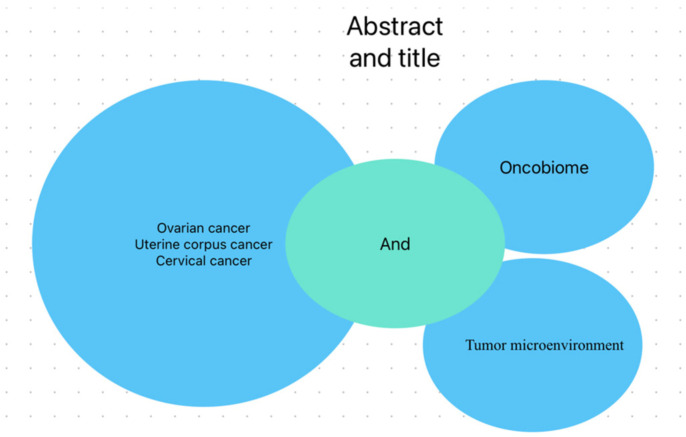
Boolean operators used in the PubMed database and Google Scholar.

A total of 5287 records were identified through PubMed and Google Scholar databases. In total, 1903 records were registered after removing duplicated records, while 119 articles were assessed for eligibility and 72 studies were included after they met the eligibility criteria. PRISMA flow diagram in Figure 1 illustrates process of selected articles. Selected phrases are presented in Figure 2.

### 3.1. Ovarian Cancer

In 2020 global new ovarian cancer incidences were 313,959, and 207,252 patients died. It is the 8th most common cancer among women [2]. Mortality is high because the disease is usually diagnosed at an advanced stage. In the early stages there are no serious symptoms of the disease. About 70% of women are diagnosed at FIGO III/IV stage [3] which indicates metastasis outside the pelvis. Despite many available ways of treatment, the 5-year survival rate is 48% [4].

The human body has its own characteristic microbiome, which contains bacteria, viruses and fungi. We could define dysbiosis as a typical flora disorder. Much scientific research described the characteristics of ovarian cancer microbiome which is connected with carcinogenesis. In the future, they could be used as a biomarker for disease detection at an early stage. Furthermore, we could use microbiota as innovative methods of ovarian cancer treatment. A remarkable fact is that the ovarian cancer microbiome is similar to that of distant organs such as head and neck cancer and triple negative breast cancer [5]. Multicenter cooperation can give a possibility for early detection and disease treatment for these malignancies.

Inside ovarian cancer cells, representatives of bacteria, fungi and viruses are observed. In this section, we are considering viruses. Banerjee S. et al.’s research discovered that *Retroviridae*, *Hepadnaviridae*, *Papillomaviridae*, *Flaviviridae*, *Polyomaviridae* and *Herpesviridae* are present in most examined ovarian cancer cells. We should especially take notice of the human papilloma virus (HPV), because there were studies which generally connected HPV not only with cervical cancer, but also with ovarian cancer [5,6]. Additionally, in healthy ovarian cells there is genetic material of HPV, although most oncogenic types (16 and 18) responsible for cervical cancer existed only in cancerous tissue. Even Merkel Cells Polyomaviruses which cause skin cancer were found in ovarian cancer cells [7]. Herpes viruses: HHV6A and HHV6B which are responsible for a three-day fever were also found in cancer cells [8]. Adenocarcinoma genetic material often contains HHV7. A few parapoxyviruses have an influence on host genetic material and force the expression of genes responsible for the synthesis of Interleukin-10, which controls inflammatory response and has an immunosuppressive effect [9]. Al-Shabanah O.A. et al. proved that in 42% of ovarian cancer samples, HPV signatures were found, and, on the other hand, only in 8% of healthy and tissues bordering a tumor. The most frequent were adequately HPV-16, HPV-18, and HPV-45 [10].

Another important aspect are bacteria. Bacterial signatures were detected in ovarian cancer tissue. There is a significant difference between the healthy environment and tumor microenvironment (TME). Per ref. [11], bacteria were detected only in tumor samples, but two types of bacteria dominated in TME. Proteobacteria were present in 52% cells and Firmicutes in 22%. Moreover, *Bacteroides*, *Actitobacteria*, *Chlamydiae*, *Fusobacteria*, *Spirochaetes* and *Tenericutes* were also found in cancer samples, albeit in a smaller percentage. In 91% of cancer samples, *Shewanella* signatures were detected. Elements of the tumor microenvironment can affect the progression of cancer, for example, *Chlamydia* inhibits the process of apoptosis. That is one of many reasons why ovarian cancer cells are immortal. This is promoting carcinogenesis, due to inhibiting mitochondrial caspase-3 [12]. As mentioned before, the connection between the microbiome of intestinal and female reproductive tract is evident. Zhou B. et al. discovered that *Proteobacteria* were detected in most ovarian cancer cells [13]. Moreover, *Proteobacteria* were found in patients with gut dysbiosis [14]. Unfortunately, there is no research which could define the risk of cancer if *Proteobacteria* in feces samples are detected. In patients with ovarian cancer, *Lactobacillus* colonies in the vaginal microbiota were decreased [15]. An early ovarian cancer marker which could be useful is the number of *Lactococcus piscium*, which were decreased in malignant cells [13]. Morikawa A. et al. discovered that the cervicovaginal microbiome of patients with ovarian cancer and with a pathogenic mutation of BRCA1, contain mostly non-Lactobacilli microbiomes [16].

Significant perturbations were found in fungal signatures. Banerjee S. et al. detected *Cladosporia* in all samples. Additionally, *Pneumocystis*, *Acremonium*, *Cladophialophora*, *Malassezia*, *Microsporidia*, and *Pleistophora* were found in a lesser concentration. In 95% of malignant cells, the genetic material of *Rhizomucor*, *Rhodotorula*, and *Alternaria* was found also. *Geotrichum* were present in healthy tissues and in tumor tissues. This could be evidence of a malignant kind of tumor. An important conclusion is that *Phaeosphaeriaceae*, which was detected in TME, reduces median progression-free survival (PFS) from 498 to 135 days [17].

Except for viruses, bacteria and fungi in TME there are representatives of parasites. In all tumor samples, *Dipylidium*, *Trichuris*, *Leishmania* and *Babesia* were mostly found [18]. In over 95% of the ovarian cancer samples, *Trichinella*, *Ascaris* and *Trichomonas* were found. Chen Y.F. et al. claimed that *Trichomonas* is responsible for the hyperproliferation of epithelium in chronic diseases. It could be connected with oncogenesis. Furthermore, *Aspergillus terreus* metabolites in the ovarian cancer TME were able to inhibit the proliferation of cancer stem cells [18].

Furthermore, lactic acid producers *Coriobacteriaceae* and *Bifidobacerium* dominated the gut microbiome of patients with platinum resistance ovarian cancer [19]. *Gammaproteobacteria* is detected in the ovarian cancer TME, and influences gemcitabine structure, causing a losing biological effect of 2nd line chemotherapy of ovarian cancer. Adding ciprofloxacin could eliminate that effect [20].

### 3.2. Cervical Cancer

In 2020, new cervical cancer incidences were 604,127 worldwide and 341,831 patients died. It is the 4th most common cancer among women [21].

After menopause, the concentration of estrogen decreases. Low levels of estrogen facilitate the development of bacteria in the vaginal microenvironment such as *Gardnerella vaginalis*, *Prevotellabivia*, *Porphyromonas*, *Sneathia*, *Leptotrihia* and *Fusobacterium*, which favors oncogenesis in cervical cells [22]. Meta-analysis conducted by Liang Y. et al. proved that the presence of vaginal bacteriosis, caused by *Candida*, *Chlamydia*, *Trachomatis* or *Ureaplasma urealyticum* is connected with HPV infection [23]. The most prevalent oncogene types of HPV are 16 and 18. Additionally, bacterial vaginosis increases the chance of cervical intraepithelial neoplasia. The presence of the infection alters vaginal pH which enables enzymes produced by HPV to damage the integrity of the cervical epithelium. The presence of *Sneathia*, which is usually present during carcinogenesis, is a marker of chronic HPV infection and cervical cancer progression. Ref. [24] in TME can be used.

In the ongoing process of carcinogenesis, raised levels of the serum of anti-inflammatory Interleukin 4 (Il-4) and Transforming growth factor β1 (TGFB1) were observed. Their presence was associated with *Fusobacterium* [25]. It also creates an immunosuppressive microenvironment, which promotes carcinogenesis.

Sims T.T. et al. compared the fecal microbiome of women with cervical cancer and healthy ones. Patients suffering from cervical cancer had an increased number of several microbiota: *Prevotella*, *Porphyromonas* and *Dialister*. On the other hand, the cervix of healthy patients had more of *Acteroides*, *Alistipes*, *Lachnospiracea* in their fecal flora. Authors claimed that the gut microbiome can induce inflammatory response through activating Toll-like receptors (TLR), because of pathogen-associated molecular patterns (PAMPs) [26].

HeLa Cells infected with *E. coli* or *Pseudomonas aeruginosa* induced the overexpression of TLR-4 receptors, which does not occur in normal keratinocytes. Most likely, they cause the downregulation of all subunits of integrins, which play a role in the carcinogenesis of the cervical epithelium. Moreover, the expression of the beta-6 subunit has a negative impact on disease [11]. An important fact about HeLa cells is that endophyte fungi from *Ginkgo biloba* produce podophyllotoxin, which stops the proliferation of HeLa cells. In this way, it promotes their apoptosis, blocks their migration and weakens the growth of HeLa cells [27].

Another scientific study proved that patients with high-grade squamous intraepithelial lesion had more bacteria connected with bacterial vaginosis, such as *Sneathia sanguinegens* and *Anaerococcus tetradius*, than patients with low-grade squamous intraepithelial lesion. Additionally, both groups were shown to have increased amounts of *Streptococcus agalactiae* and *Clostridium* [28].

The biggest threat of cervical cancer metastasis exists when *Klebsiella* and *Robignitomaculum* are in TME, and the lowest risk exists when *Microminisoira* and *Kobuvirus* are in TME [29]. Authors of the same research classified cervical cancer in terms of the microbiome. Overall survival (OS) depends on five bacteria: *Methylobacter*, *Robignitomaculum*, *Klebsiella*, *Micromonospora* and *Microbispora*. *Methylobacter* has a harmful impact on mortality, while the remaining ones have a positive impact on survival. Cervical cancer cells infected with *Methylobacter* had a higher expression of CTLA-4 and PD-1 than cells infected with *Robignitomaculum*, *Klebsiella*, *Micromonospora* and *Microbispora*. For the first group of patients, immunotherapy could be a possible treatment. Routy B. et al. proved that the composition of the gut microbiome has an influence on cancer treatment, immune resistance, and efficiency of immunotherapy [30].

It is noteworthy that the microbiome does not only have negative sides. It can be used as a treatment. Fan Q. et al. proved that lactic acid produced by *Lactobacillus iners* also activates the Wnt pathway. Consequently, the fucosylation of epithelial cells stops the proliferation and migration of cervical cancer cells [31]. Zhong K. et al. discovered that schizophyllan (SPG) produced by fungi *Schizophyllum* has anticancer properties [32]. *Rhabdoviridae*, as an oncolytic virus, is responsible for cancer cell apoptosis. Cervical cancer tumors, in which cells were infected with *Rhabdoviridae*, have decreased [33].

### 3.3. Uterine Corpus Cancer

The incidences of uterine corpus cancer were 417,367, and 97,370 patients died. It is the 6th most common cancer among women. Worldwide per 100,000 individuals, Poland had the highest rate of endometrial cancer in 2020 [34].

The most common risk factor for uterine corpus cancer is obesity, disbalance between estrogens and progesterone or chronic endometritis. Changes in the metabolism of estrogens are connected with the dysbiosis of vaginal and gut microbiome. It was proved that there exists a connection between dysbiosis of the female reproductive tract, chronic expression of inflammatory cytokines and endometrial cancer. Kuźmycz O et al. proved that the microbiome of endometrial cancer is more diversified [35]. Yang, Y. et al. constructed a prognostic model based on the Resident Microbiome of Endometrium (RME) [19]. They assigned an RME score to the risk of death. Based on their research, patients with endometrial cancer in whose microbiome *Zooshikella*, *Caldimonas*, *Candidatus*, *Accumulibacter*, *Nonlabens*, *Roseiflexus*, *Streptosporangium*, *Zavarzinellaa* were found had better overall survival (OS). On the contrary, *Derxia* and *Shigella* worsened OS. Among them, *Shigella* is heavily connected with endometrial cancer. It was reported that there is a difference between the microbiome of upper and lower female genitalia in women who had a hysterectomy for malignant disease, hyperplasia or benign disease [36]. When observing the microbiome of the vagina, cervix, fallopian tubes and ovaries in patients with uterine corpus cancer, it was found that they have more bacteria, such as Firmicutes (*Anaerostipes*, *Dialister*, *Peptoniphilus*, *Ruminococcus*, *Anaerotruncus*), Spirochaetes (*Treponema*), *Bactreroides*, *Arthrospira* and *Faecalbacterium*, than other female reproductive tract organs. Other microbiomes which increase the risk of uterine corpus cancer are *Atopobium vaginae* and *Porphyromonas*. These bacteria stimulate endometrial cells to secrete inflammatory substances such as IL1α, IL1β, IL17α and TNFα. They maintain the process of neoplasia [37,38,39]. In the first stage, they cause hyperplasia followed by dysplasia of uterine corpus epithelium. Moreover, more *Actinobacteria*, *Firmicutes*, *Proteobacteria* and *Bacteroides* were found in tissue samples of patients with obesity than in patients with correct Body Mass Index (BMI) [40]. Microbiome diversity also depends on the patient’s race. Actinobacteria and Firmicutes occurred frequently in Afro-American patients with endometrial cancer. An abundance of *Anaerococcus* in the microbiome of endometrial cancer was an important factor separating endometrial and non-endometrial cancer. Moreover, *Anaerococcus* raised the level of reactive oxygen species (ROS) in fibroblast inside the uterus. ROS destroy proteins, membranes, organelles and lipids. Further research on ROS and endometrial cancer should be performed [18].

In patients with chronic endometritis and improper uterine bleeding, a predominance of *E. coli*, *Staphylococcus* and *Enterococcus* were observed [38]. This is similar to cervical cancer activating TLR-4 receptors to induce proliferation and activating immune cells. As a result, chronic inflammation is detected in the endometrial microenvironment. Finally, progression to cancer occurs. As we know, endometrial cancer is divided into two types: the 1st type, endometrial cancer (80–90% cases), and the 2nd type, non-endometrial (10–20% cases). It was discovered that all patients with the 2nd type of endometrial cancer had Porphyromonas somerae and only 50% of patients with that bacteria had endometrial hyperplasia [41].

On the other hand, products of bacterial metabolism can stop the growth of endometrial tumor through inhibiting histone deacetylase [42]. Furthermore *Ruminococcus*, *Prevotella* and *Anaerostipes caccae* increase the level of long chain fatty acids (LCFA), especially C16:1 and C2:2. This can stimulate the process of proliferation and risk of metastasis through activating the mTOR pathway [43]. Moreover, Yang, Y. et al. discovered that patients with low RME scores had an elevated expression of MSI and PD1. This probably affects the response to immunotherapy [19]. In summary, endometrial hyperplasia or endometrial cancer could be differentiated in the early stage of the disease, using the elements of microbiome. In Table 1 we presented most common parts of gynecologic cancer oncobiome.

## 4. Discussion

It is important to understand the exact role of the microbiome because it creates the microenvironment of cancer. It is an enclave, because the habitat inside of the tumor allows bacteria to survive. The Cancer Genome Atlas (TCGA) created by Narunsky-Haziza L et al. defined the genome of 33 cancers [17]. A similar library of cancer microenvironments could be used as a biomarker or for oncological treatment.

A few researchers claim that the microbiome is a forgotten organ. It is estimated that the ratio between bacteria and human cells is about 1:1–1.3 [44]. Several studies discovered a connection between the microbiome, oncobiome and oncogenesis [45]. Chronic dysbiosis can lead to a chronic inflammatory process and that process could lead to oncogenesis. Processes of aging, genetic defects, antibiotics use, or lifestyle cause human microbiome disorders. In particular aging has a proinflammatory effect on the microbiome [46]. Microbiomes of tissues which are adjacent to the tumor are similar to the tumor tissues. This is caused by the response of immune cells, fibrosis and inflammation [47]. Key carcinogenesis factors are metabolic products which increase the risk of cancer or genotoxic substances (for example, colibactin produced by *E. coli* [48]). Multiple pieces of evidence prove that pathogen-associated molecular patterns (PAMP) combine—lipopolysaccharide-fatty acids and Toll-like receptors (TLRs) affect each other and form a microbiota–cancer axis. Kau A.L. et al. claim that the microbiome has a remote influence on congenital immune response and immunotherapy during cancer treatment [49].

The female reproductive tract (FRT) microbiome together with the gastrointestinal tract (GT) and urinary tract (UT) create, respectively, the vagina–gut axis and the vagina–urinary tract axis [50,51]. Furthermore, the microbiome which produces β-glucuronidase is able to metabolize estrogens. On top of that, intestinal microbiota and their genes, which are able to metabolize estrogens, are named estrobolome [52,53].

The vaginal microbiome in women of reproductive age is low-differentiated and it is based on different types of *Lactobacillus*. D’Antonio DL et al. believe that the dominance of *Lactobacillus crispatu* is the most favorable vaginal microbiome [53]. *Lactobacillus* produces lactic acid, which secures a proper level of pH. However, the composition of the vaginal microbiome is correlated with the level of estrogens. It is the principal cause of vaginal microbiome dynamism. Lactic acid creates an acid environment, which prevents pathogens from invading. Additionally, *Lactobacillus* produces bacteriocins, which kill pathogens responsible for Sexually Transmitted Diseases (STD) such as *Neisseria gonorrhoeae* [54], *Chlamydia trachomatis* [55] and *E. coli* [56]. Biofilm produced by *Lactobacillus* gasseri blocks adhering *Candida* fungus [57]. The dysbiosis of the vaginal microbiome may provoke the invasion of *Staphylococcus*, *Streptococcus* or *Enterobacteriaceae* [58] which are responsible among other pelvic inflammatory disease, for endometritis or gynecologic cancers [59].

The estimated number of bacteria in the uterus is 10 k times lower than in the vagina [60]. Before puberty and after menopause, the vagina microbiome is deprived of *Lactobacillus*. It is dominated by anoxygenic bacteria like *Anaerococcus vaginalis* [61]. On the other hand, during pregnancy, when the level of estrogen is high, the physiological vagina microbiome mainly contains *Lactobacillus crispatu* and *Iners*. The upper female reproductive tract microbiota is more diverse than the lower one [62]. Given the remarkable dynamism observed within FRT microbiota, it is difficult not to perceive this as a promising area for further scientific investigations, particularly with regard to the pathogenesis of diseases in this environment, including malignancies.

Microbiomes which are part of the vaginal microbiome are often responsible for cervical cancer, corpus uterine cancer or ovarian cancer. We cannot forget that genetic instability affects pathologic changes in DNA due to metabolic products of the vaginal microbiome. The tumor microenvironment has contact with nearby and even distant cells through lymphatic and circulatory systems. It can affect adjacent tissues and also distant organs, for example, estrogens are responsible for breast and uterine cancer [53,63]. Whether the microbiome plays a role in these processes requires further investigation.

The tumor microenvironment (TME) plays an important role in the survival and progression of cancer cells. This habitat is an inseparable part of the tumor. Data from studies confirmed that malignancies have their specific microenvironment, and it was observed that the intratumor microbiome contains cells of specific cancer and host immune cells [64]. TME provides tumor cells with nutrients which are essential for survival and proliferation [65]. It has a fundamental influence on oncogenesis regulation, immune system response and influence of microbiome metabolic products on the immune system.

Inside of the tumor microenvironment, we can distinguish the extracellular matrix (ECM), which is defined as a very dynamic, non-cellular element of TME. ECM mainly contains collagen, fibronectin, and laminin. The process of migration and proliferation of cancer cells mainly take part in the ECM [66]. Henke E. et al. proved that 60% of solid tumor mass may consist of ECM [67]. There are cancer-associated fibroblasts (CAFs) which are involved in oncogenesis, angiogenesis and cancer progression. Moreover, cancer-associated fibroblasts take part in the resistance of breast cancer to anticancer treatment [68]. It was confirmed that cancer-associated fibroblasts directly stop antitumor T-cells [27].

The interaction between TME, TME elements and cancer cells has a fundamental role in understanding and learning new and pivotal information which allows us to define the risk of metastasis and choose more accurate, individualized treatment. This also means more effective treatment.

As we know, gynecologic cancers (ovarian cancer, cervical cancer or uterine corpus cancer) are characterized by their specific microbiome. It is proved that there exists an influence of the microbiome of the female reproductive tract, gastrointestinal tract and urinary tract on carcinogenesis. It is named the vagina–gut axis or vagina–urinary tract axis [50,51]. The dysbiosis of the vaginal microbiome, especially lack of *Lactobacillus*, leads to an increased number of *Staphylococcus*, *Streptococcus* or *Enterobacteriaceae* [58], which are responsible for pelvic inflammatory disease, endometritis or gynecologic cancers [59]. Carcinogenesis starts as a vaginal dysbiosis which is caused by certain products of bacterial metabolism.

Every tumor has its specific microenvironment (TME). The three gynecologic cancers described in this article have their unique microenvironment. TME provides cancer with nutritional elements which are necessary for two basic functions: survival and proliferation [65]. To summarize this finding, it is suggested that TME supports cancer cell survival, regulates carcinogenesis and inhibits immune system response. There is a need for more studies on TME of every gynecologic cancer to define the risk of metastasis and to choose individualized treatment.

Ovarian cancer is usually diagnosed at the advanced stage. There are no effective methods of screening. The TME of ovarian cancer cells houses characteristic pathogens which cannot be found inside healthy cells [5]. More attention is required for most oncogenic types of HPV, 16 and 18. They are most frequently connected with cervical cancer, but their genetic material was found inside the TME of ovarian cancer cells [5].

Nowadays, radical surgeries in the course of cervical cancer treatment are being gradually replaced. HPV infection, especially types 16 and 18, induce vaginal microbiome dysbiosis. In the next step, vaginal microbiome disorders promote the process of neoplasia. Generally, in the TME of cervical cancer cells, *Sneathia* bacteria exist, which were found in chronic HPV infection and during oncogenesis. Further studies are needed to confirm if *Sneathia* could be used as a marker for cervical cancer detection. Additionally, Fusobacterium creates an immunosuppressive microenvironment, which simplifies the proliferation of cervical cancer cells [25]. There are specific bacteria which are connected to overall survival and risk of metastasis. Cervical cancer cells that include *Methylobacter* in their TME are thought to have a better response to immunotherapy. This is due to their higher expression of PD-1 and CTLA-4 receptors.

Both morbidity and mortality due to uterine corpus cancer are rising. In 2013, the molecular classification of endometrial cancer was published. The molecular type of endometrial cancer is important for prognosis and treatment. More precise treatment can be introduced through elements of the microenvironment. Inside endometrial cells, *Atopobium vaginae* and *Porphyromonas* stimulate the secretion of proinflammatory cytokines: IL1α, IL1β, IL17α and TNFα [39]. It has been shown that these particular patients have increased amounts of *E. coli*, *Staphylococcus* and *Enterococcus* [38]. Nevertheless, this topic is still under investigation.

To summarize, further research on the microbiome and TME of gynecologic cancers is needed, i.e., how to use elements of the gynecologic cancer microbiome and TME for their diagnosis and treatment. In conclusion, additional studies are necessary to deepen our understanding of the microbiome and TME in gynecologic cancers. It is imperative to investigate new elements of the microbiome, and TME can be utilized to enhance the diagnostic accuracy and therapeutics strategies for these cancers. Additionally, exploring the interplay between these factors may provide valuable insights into personalized treatment and approaches, potentially improving patient outcomes and advancing clinical practices in gynecologic cancers.

## 5. Conclusions

The tumor microenvironment is very varied. The oncobiome can be used for the diagnosis of cancer. It can be used as a target to create an atlas of the oncobiome of all known cancers. This type of atlas could be used as a pattern for diagnosis. For example, we could systematically check the uterine corpus oncobiome of patients with BRCA1 or BRCA2 mutation or with Lynch Syndrome. The next step will be to discover how to use TME as a target for cancer therapy. New research should be performed to discover how doctors could use them as a method of treatment. The oncobiome can be used as a part of treatment. Some patients will respond better to immunotherapy, and some to “oncobiometherapy”.

## Figures and Tables

**Table 1:** Most common oncobiome in gynecologic cancer.

	Bacteria	Viruses	Fungi and Parasites
**Ovarian cancer**	*Proteobacteria* *Firmicutes*	*Retroviridae*, *Hepadnaviridae*, *Papillomaviridae*, *Flaviviridae*, *Polyomaviridae* and *Herpesviridae*	*Cladosporia* *Dipylidium*, *Trichuris*, *Leishmania* and *Babesia*
**Cervical cancer**	*Gardnerella vaginalis* *Prevotellabivia* *Sneathia*	HPV	*Schizophyllum*
**Uterine corpus cancer**	*Shigella*	**-**	**-**

## Abbreviations

The following abbreviations are used in this manuscript:

FIGO	The International Federation of Gynecology and Obstetrics
HPV	Human Papilloma Virus
HHV	Human Herpesvirus
TME	Tumor Microenvironment
Il	Inflammatory interleukin
TGFβ1	Transforming growth factor β1
TLR	Toll-like receptors
PAMPs	pathogen-associated molecular patterns
OS	Overall Survival
RME	Resident Microbiome of Endometrium
ROS	reactive oxygen species
TNF	Tumor necrosis factor
LCFA	long chain fatty acids
TCGA	The Cancer Genome Atlas
FRT	Female reproductive tract
GT	gastrointestinal tract
STD	Sexually Transmitted Diseases
ECM	extracellular matrix
